# Diagnostic Accuracy of Procalcitonin Compared to C-Reactive Protein and Interleukin 6 in Recognizing Gram-Negative Bloodstream Infection: A Meta-Analytic Study

**DOI:** 10.1155/2020/4873074

**Published:** 2020-01-23

**Authors:** Liying Lai, Yijie Lai, Hao Wang, Liang Peng, Ning Zhou, Yi Tian, Yongfang Jiang, Guozhong Gong

**Affiliations:** ^1^Department of Infectious Diseases, The Second Xiangya Hospital, Central South University, Changsha 410011, China; ^2^Department of Functional Neurosurgery, Ruijin Hospital, Shanghai Jiao Tong University School of Medicine, Shanghai 200025, China; ^3^Department of Pharmacology, Shanghai Jiao Tong University School of Medicine, Shanghai 200025, China; ^4^Department of Infectious Diseases, 3rd Affiliated Hospital of Sun Yat-sen University, Guangzhou 510000, China

## Abstract

**Objective:**

Gram-negative bloodstream infections (GNBSIs), especially those caused by antibiotic-resistant species, have become a public health challenge. Procalcitonin (PCT) showed promising potential in early diagnosis of GNBSI; however, little was known about its performance under different clinical settings. We here systematically assessed the diagnostic accuracy of PCT in recognizing GNBSI and made direct comparisons with C-reactive protein (CRP) and interleukin 6 (IL-6).

**Methods:**

PubMed, Embase, ISI Web of Knowledge, and Scopus were searched from inception to March 15th, 2019. Area under the summary receiver operating characteristic curve (AUC), pooled sensitivity, specificity, and diagnostic odds ratio (DOR) were calculated. Hierarchical summary receiver operating characteristic (HSROC) model was used for the investigation of heterogeneity and for comparisons between markers.

**Results:**

25 studies incorporating 50933 suspected BSI episodes were included. Pooled sensitivity and specificity for PCT were 0.71 and 0.76, respectively. The overall AUC was 0.80. The lowest AUCs were found in patients with febrile neutropenia (0.69) and hematological malignancy (0.69). The highest AUC was found in groups using electrochemiluminescence immunoassay (0.87). In direct comparisons, PCT showed better overall performance than CRP with the AUC being 0.85 (95% CI 0.81–0.87) for PCT and 0.78 (95% CI 0.74–0.81) for CRP, but the relative DORs varied with thresholds between PCT and CRP (*p* < 0.001). No significant difference was found either in threshold (*p* < 0.001). No significant difference was found either in threshold (*p* < 0.001). No significant difference was found either in threshold (

**Conclusions:**

PCT was helpful in recognizing GNBSI, but the test results should be interpreted carefully with knowledge of patients' medical condition and should not serve as the only criterion for GNBSI. Further prospective studies are warranted for comparisons between different clinical settings.

## 1. Introduction

Gram-negative bloodstream infection (GNBSI) is a common type of bacterial infection and also the leading cause of septic shock [1]. Missed identification of GNBSI delays treatment, increasing the risk of disability and mortality. On the other hand, the overuse of antibiotic agents in patients without GNBSI usually leads to antibiotic resistance. GNBSI caused by antibiotic-resistant species has become a public health challenge with substantial morbidity and mortality [2, 3]. Therefore, early diagnosis of GNBSI is crucial for disease management. Blood culture is the gold standard in identifying causative pathogens for bloodstream infection (BSI); however, standard incubation processes would take nearly 5 days and false negatives often occur [4]. Though advanced techniques were proposed for pathogen identification, including high-throughput polymerase chain reaction (PCR), microarray-based assays, and matrix-assisted laser desorption ionization–time of flight mass spectrometry (MALDI-TOF MS), their demands for skills and equipment were too strict to be widely satisfied, especially in less developed regions [5–7].

Procalcitonin (PCT), a 116 amino acid peptide biomarker, has been extensively investigated in differentiation between bacterial infection and systematic inflammatory response syndrome (SIRS) of noninfectious origin [8]. Recent studies suggested that highly elevated blood PCT level was associated with Gram-negative infection [9]. In healthy volunteers, PCT was found to increase within 4 hours after the injection of endotoxin, a specific pathogenic factor of Gram-negative bacteria, and fall rapidly during recovery [10]. This feature makes PCT an ideal candidate for early identification of GNBSI with further potential in guiding antibiotic treatment. Some studies have compared PCT with its counterparts which also exhibit potential in recognizing GNBSI, e.g., C-reactive protein (CRP) and interleukin 6 (IL-6) [9]. However, the results of these comparisons were inconsistent, and the patients' medical conditions varied greatly between studies [9].

So far, the value of PCT in early identification of GNBSI is still argued by researchers and is poorly explored in guidelines [11]. Two meta-analyses on this topic were published before, but their clinical utility was limited by either poor investigation of underlying heterogeneity or not investigating the proper diagnostic indices [12, 13]. Therefore, we herein systematically assessed the diagnostic accuracy of PCT in recognizing GNBSI in patients with suspected BSI and examined the factors associated with threshold and diagnostic accuracy. We also made direct comparisons between PCT and other markers showing potential in recognizing GNBSI, including CRP and IL-6.

## 2. Materials and Methods

This meta-analysis was conducted in accordance with the Cochrane Collaboration's Diagnosis Test Accuracy Working Group protocol [14]. Findings were reported following the Preferred Reporting Items for Systematic Reviews and Meta-Analyses (PRISMA) guideline (Supplementary [Supplementary-material supplementary-material-1]) [15]. The protocol was registered with the PROSPERO database (registration number CRD42018092664).

### 2.1. Search Strategy and Selection Criteria

Databases including PubMed, Embase, ISI Web of Knowledge, and Scopus were searched from inception to March 15th, 2019. The searched Mesh terms (for Medline), EMTREE terms (for Embase), and text words (for others) were “(procalcitonin OR PCT) AND (bloodstream infection OR BSI OR bacteremia) AND (sensitivity OR specificity OR diagnose OR predict) AND Gram negative”. Reference lists of previous reviews and included original articles were also checked.

Studies were independently reviewed by two investigators (YL and NZ). Eligible studies should (1) assess the diagnostic accuracy of PCT in recognizing GNBSI in a context of suspected bloodstream infection (BSI), (2) provide a clear culture result, and (3) written in English. The exclusion criteria were (1) animal experiments, reviews, case reports, conference abstracts, and expert opinions; (2) information insufficient for calculating the number of true positives, false positives, false negatives, and true negatives; (3) analysis with mixed culture results; and (4) case-control studies with healthy controls. In comparisons between markers, heterogeneity in the estimated accuracy of a diagnostic test across studies is likely to occur and would confound the comparisons. Therefore, in comparing the performance between markers, we only included studies that made a direct comparison of the tests of interest either by applying both tests to each individual or by randomizing each individual to receive one of the tests [14].

### 2.2. Data Extraction

Two investigators independently extracted the following data: author, year, region, assay methods for PCT, cutoffs, study design, settings, true positives, false positives, false negatives, and true negatives. Since there were no established criteria for the optimal cutoff in this diagnostic theme and the proposed optimal cutoff varied greatly between studies, we extracted the data with the highest Youden's index if multiple cutoffs were presented in a study for the index test. We referred to the corresponding authors if further information was needed.

### 2.3. Quality Assessment

Methodological quality of the studies was assessed using the Quality Assessment of Diagnostic Accuracy Studies 2 (QUADAS-2) [14]. Modifications and redefinitions were made to the rules in the QUADAS-2 tool as described in Supplementary Tables [Supplementary-material supplementary-material-1] and [Supplementary-material supplementary-material-1]. The assessment was performed independently by two authors (YL and HW). Discrepancies were resolved in a consensus meeting.

### 2.4. Statistical Analysis

Bivariate mixed-effects regression model was used to calculate the pooled estimates of sensitivity, specificity, and diagnostic odds ratio (DOR) with their standard errors and 95% CIs. Hierarchical summary receiver operating characteristic (HSROC) curves were constructed to assess the overall diagnostic performance. The area under the summary receiver operating characteristic curve (AUC) was used to reflect the overall predictive power. The unit of the primary analysis of this review is a suspected BSI episode. As the optimal cutoffs varied greatly from 0.291 ng/mL to 16.9 ng/mL among the included studies, we used the scatter of points and prediction ellipse to depict the observed heterogeneity graphically [14].

The direct comparisons were implemented by Rutter and Gatsonis HSROC model. We also explored the effect of covariates on heterogeneity in test thresholds (or cutoff values) and diagnostic accuracy with this model [14]. In metaregression, a *p* value based on the likelihood ratio *χ*^2^ statistic was calculated. The *χ*^2^ statistic is computed as the change in the –2Log likelihood when a covariate is added (or removed) from the logistic regression model. When statistical significance is found in a test threshold between two and three conditions of a certain covariate, it is suggested that the SROC curves of these conditions have different shapes and the ratio of diagnostic odds ratio (rDOR) will not be constant along the entire length of the curve, which means the relative diagnostic accuracy under these different conditions varies with thresholds [14]. If no statistical significance was found in the test threshold, then the HSROC model could be further simplified by removing the parameters for threshold, leaving only parameters for accuracy [14]. Investigated covariates can be classified into three main categories, namely, (1) covariates of medical contexts, (2) covariates of demographical features, and (3) other covariates. Covariates of medical contexts included type of BSI (only in Gram-negative and Gram-positive BSI or not), sepsis status (only in sepsis patients or not), hematological malignancy status (only in patients with hematological malignancy or not), febrile neutropenia status (only in febrile neutropenic patients or not), and culture (only in positive cultures or not); covariates of demographical features included region (east Asia or Europe), setting (only in ICU or not), and population (only in adult population or not); other covariates included assay method for PCT (BRAHMS-VIDAS, BRAHMS-KRYPTOR, or electrochemiluminescence immunoassay) and sample type (serum or plasma). Fagan nomogram was made to determine the posttest probabilities [16]. Deek's funnel plot was drawn to assess the publication bias [14].

## 3. Results

### 3.1. Study Search and Selection

**Figure 1:**
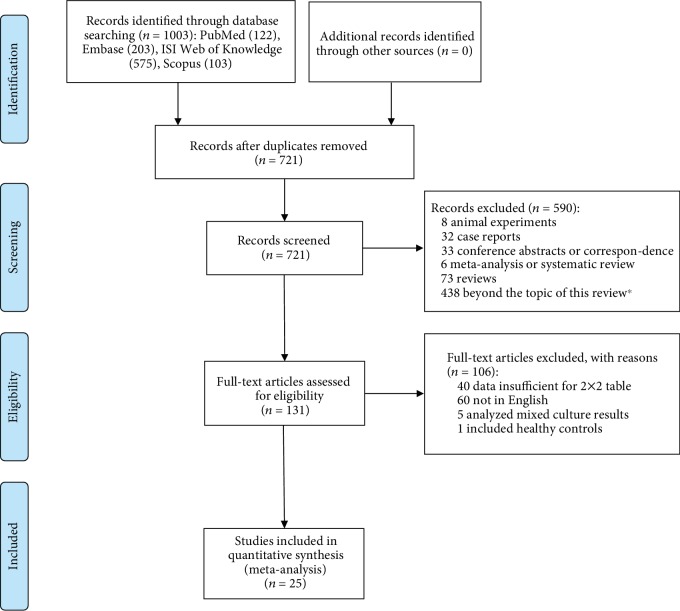
Study selection. ^∗^Beyond the topic of this review: once the article types were qualified, studies were further checked for their topic; ineligible studies were excluded.

Shown in Figure 1, the search retrieved 1003 records. After screening titles and abstracts, 131 full-text articles were assessed and 25 were included [17–41]. If PCT was used to discriminate GNBSI from two different types of BSI with overlapped population in a study, datasets with the largest sample number were adopted [26, 27, 36].

### 3.2. Study Characteristics

Following our inclusion criteria, this present study included 25 studies with 50933 suspected BSI episodes from over 45576 patients. Main characteristics of these studies were shown in Table 1 and Supplementary [Supplementary-material supplementary-material-1]. Among these episodes, 4544 (8.9%) were confirmed as GNBSI. The median prevalence of GNBSI in patients with suspected BSI across the included studies was 46.99% (IQR 16.78%-53.97%), with the lowest prevalence being 3.02% and the highest being 71.82% [32, 37]. 16 (64%) studies were retrospectively designed. 18 (72%) studies only included adult patients. All included studies used blood culture as the reference standard. Quantitative PCT assay was the index test, and the most commonly used assay technique was BRAHMS-VIDAS (Supplementary [Supplementary-material supplementary-material-1]).

### 3.3. Quality Assessment

The overall and individual dataset's quality assessment according to our tailored QUADAS-2 checklist in four domains (“patient selection,” “index test,” “reference standard,” and “flow and timing”) are summarized in Supplementary Figures [Supplementary-material supplementary-material-1] and [Supplementary-material supplementary-material-1]. All included studies used blood culture as the reference standard for diagnosis of GNBSI. In general, the included studies showed moderate (without high-risk items) risk of bias in three of the four domains and high applicability, but high risk of bias in “index test” domain was found in 11 studies [17–23, 28, 30, 31, 33]. The high risks of bias were mainly caused by using a data-driven method, namely ROC analysis, for calculation of optimal cutoff in a relatively small number of patients [42].

### 3.4. Diagnostic Accuracy of PCT

**Figure 2:**
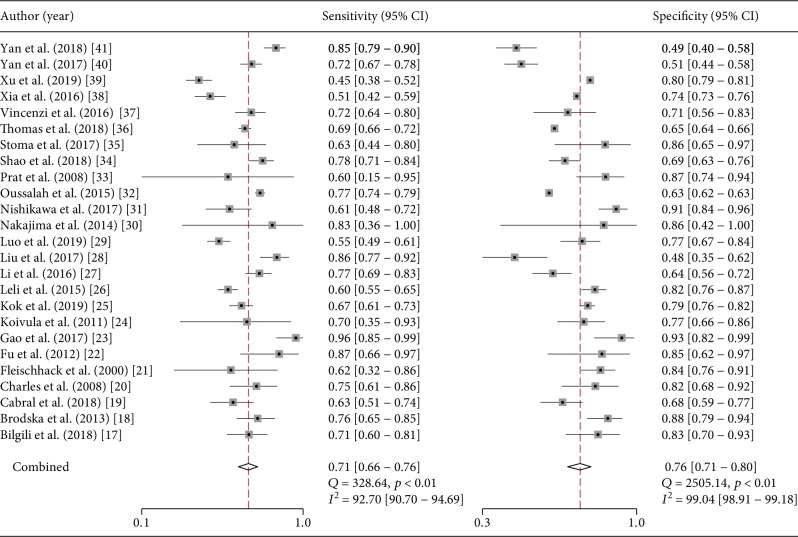
Pooled sensitivity and specificity of PCT for recognizing GNBSI in patients with suspected bloodstream infection (BSI).

**Figure 3:**
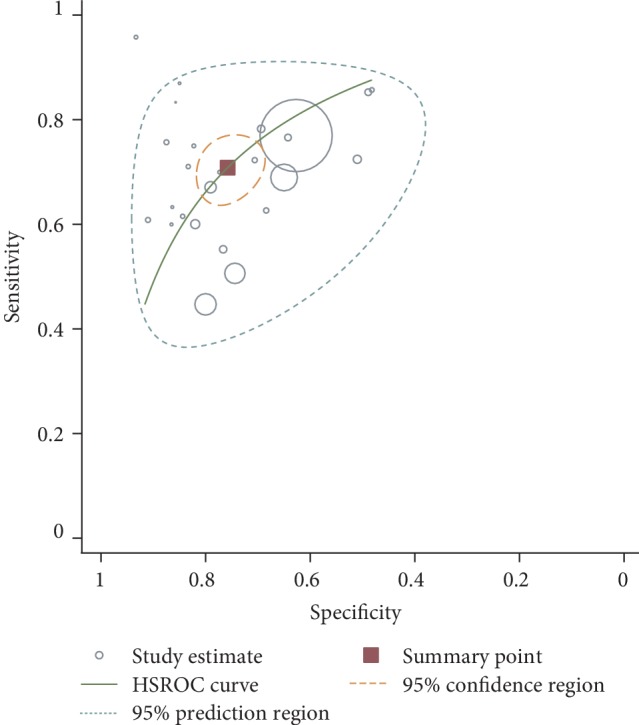
Hierarchical summary receiver operating characteristic (HSROC) curve of PCT for recognizing GNBSI in patients with suspected bloodstream infection (BSI).

For recognizing GNBSI in a context of BSI, the median optimal cutoff value of PCT was 1.3 (IQR 0.5-8.06) ng/mL, the pooled sensitivity and specificity were 0.71 (95% CI 0.66-0.76) and 0.76 (95% CI 0.71-0.80) (Figure 2), respectively, and the pooled DOR was 7.60 (95% CI 5.51-10.48) (Supplementary [Supplementary-material supplementary-material-1]). The value of AUC was 0.80 (95% CI 0.76-0.83) (Figure 3). As substantial heterogeneity was indicated by the scatter of points and prediction ellipse, we further conducted subgroup and metaregression analysis. In the subgroup analysis, the lowest values of AUC were found in patients with febrile neutropenia (0.69) and hematological malignancy (0.69), and the highest value of AUC was found in groups using electrochemiluminescence immunoassay (ECLIA) (0.87). The lowest sensitivity was found in patients with hematological malignancy (0.52); the highest sensitivity was found in discriminating GNBSI from Gram-positive BSI (0.77). The lowest specificity was found in groups using BRAHMS-KRYPTOR assay (0.65); the highest specificity was found in groups using ECLIA (0.84) (Table 2).

**Figure 4:**
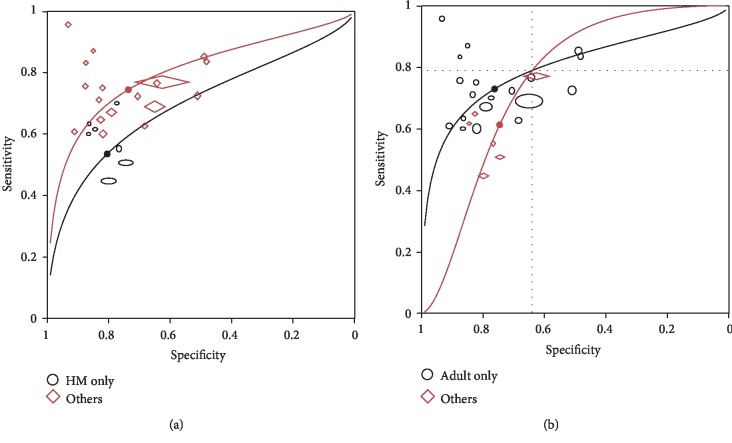
Comparisons of diagnostic accuracy of PCT in population with different (a) hematological malignancy statuses (only in patients with hematological malignancy or not) and (b) ages (only in adults or not). HM: hematological malignancy. Sizes of circles and diamonds represent relative sample sizes in each study.

In analyzed covariates of medical contexts, diagnostic accuracy of PCT was found not to vary with thresholds (*p*1 > 0.05). With further simplification of the model, the diagnostic accuracy of PCT was found significantly lower in hematological malignancy patients (*p*2 = 0.032, Figure 4(a)). In the comparison between studies with adult population and mixed population (adult and pediatric patients), the rDOR of PCT was suggested to vary with thresholds (*p*1 = 0.043, Figure 4(b)). No statistically significant impact of the rest of the investigated covariates, including types of BSI, sepsis status, febrile neutropenia status, culture positivity, region, settings, assay method for PCT, and sample type, was found either on threshold or on accuracy (*p*1 > 0.05, *p*2 > 0.05, Table 2).

Supposing the pretest probability of GNBSI in all patients with suspected BSI to be 47% (the median prevalence of GNBSI in patients with suspected BSI), Fagan's nomogram for likelihood ratios indicated that, with the assistance of PCT test, the postprobability increased to 72% when the PCT test results were positive and the postprobability decreased to 25% when the results were negative (Supplementary [Supplementary-material supplementary-material-1]) [16]. Deek's funnel plots suggested potential publication bias (*t* = 2.48, *p* = 0.02, Supplementary [Supplementary-material supplementary-material-1]).

### 3.5. Comparisons of PCT with CRP and IL-6

In 13 studies simultaneously assessing the performance of CRP and PCT for discriminating GNBSI from BSI of other origins in a total of 7371 episodes, the pooled DORs of PCT and CRP were 11.40 (95% CI 6.13–21.21) and 6.39 (95% CI 3.40-11.99) (Table 3). In 5 studies simultaneously assessing the performance of IL-6 and PCT in a total of 3455 episodes, the pooled DORs of IL-6 and PCT were 11.86 (95% CI 3.95-35.64) and 17.98 (95% CI 4.47–72.41). Additionally, these later five studies also investigated the performance of CRP with a pooled DOR being 11.86 (95% CI 3.29–42.74).

**Figure 5:**
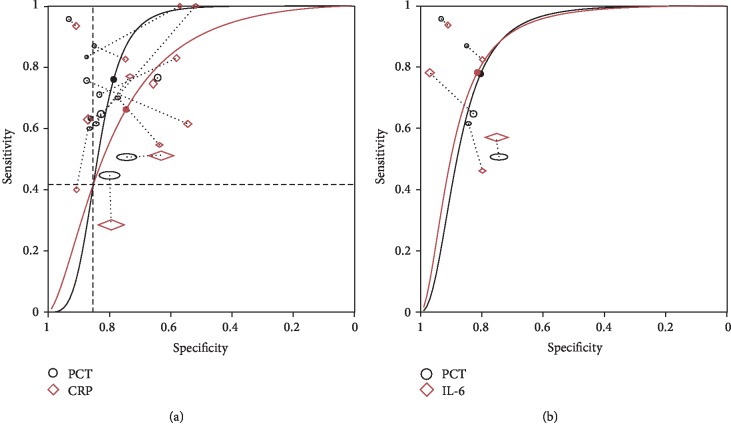
Direct comparisons between PCT and CRP (a), and between PCT and IL-6 (b). Observations connected by dash lines were reported in the same study. Sizes of circles and diamonds represent relative sample sizes in each study.

In direct comparisons between biomarkers, PCT showed higher overall performance than CRP with the AUC being 0.85 (95% CI 0.81–0.87) for PCT and 0.78 (95% CI 0.74–0.81) for CRP. However, the shape of the summary curve differed between studies using PCT and CRP (*χ*^2^ = 446.4 − 434.2 = 12.2, *p* < 0.001), which indicated that the relative accuracy of the test would vary with threshold (Figure 5(a)). Focusing on the region of the plot covering the observed data, the interpretation of which marker showed higher accuracy depended on the threshold: when the specified threshold defined a sensitivity > 0.42 or a specificity < 0.85, the diagnostic accuracy was higher in PCT test compared to CRP [14]. In the comparison between PCT and IL-6, the two curves can be assumed to have the same shape (*χ*^2^ = 125.2 − 125 = 0.2, *p* = 0.654), indicating the relative accuracy would not vary with thresholds (Figure 5(b)). Though bivariate model showed a higher diagnostic odds ratio in PCT than in IL-6, further simplification of the HRSOC model showed no significant difference in diagnostic accuracy between PCT and IL-6 (*χ*^2^ = 125.7 − 125.2 = 0.5, *p* = 0.480).

## 4. Discussion

Recent original studies and meta-analyses highlighted the effectiveness of PCT protocols in early diagnosis of bacterial infection and further in assisting in the initiation and termination of antibiotic treatment [43–47]. Though the value of PCT in recognizing GNBSI has been explored, utility of the results in most studies is hampered by either small sample size or limited clinical information. Only two meta-analyses were published on this topic [12, 13]. He et al. estimated the overall accuracy of PCT for diagnosing GNBSI and found its sensitivity being 0.73 (95% CI 0.68 to 0.78), specificity being 0.74 (95% CI 0.64 to 0.81), DOR being 7.59 (95% CI 5.31 to 10.85), and AUC being 0.79 [13]. In their study, pairs of sensitivity and specificity were transformed into a single indicator (diagnostic odds ratio) to investigate heterogeneity; as a result of this process, the analysis was simplified but the merits of the two-dimensional nature of the data were lost [48]. Furthermore, the analyzed covariates were so limited that the difference between specific conditions, including age, background diseases, and PCT test methods, could not be revealed. In the other meta-analysis, Tang et al. compared concentrations of PCT in patient with Gram-negative and Gram-positive bloodstream infections; however, the diagnostic indices, such as sensitivity and specificity, were not investigated [12].

The results of this meta-analysis indicated a helpful potential of PCT in recognizing GNBSI with an overall AUC of 0.80. This diagnostic value maps onto an increase to 72% in positive postprobability and a decrease to 25% in negative postprobability compared to a pretest probability of GNBSI of 47%. The relative diagnostic value varied between different patient populations with AUC values ranging from 0.69 in febrile neutropenia and hematological malignancy patients to 0.87 in groups using electrochemiluminescence immunoassay. To our knowledge, this is the first meta-analysis to provide direct comparisons of the diagnostic value of PCT with CRP and IL-6 in recognizing GNBSI. We herein identified a trend indicating PCT being superior to CRP in recognizing GNBSI, while the relative diagnostic ratio changes across thresholds.

### 4.1. Factors Influencing the Performance of PCT

Different pathogens are believed to induce varied levels of PCT as they activate different Toll-like receptor signaling pathways [49]. In healthy individuals, PCT found in the circulation would be ≤0.1 ng/mL [50]. Normal or slightly elevated PCT level in critically ill septic patients was more likely to be a result of viral infection or systemic inflammatory response of noninfectious origin rather than bacteremia (including both Gram-negative and Gram-positive infection) or fungemia [12, 22, 48, 51]. In a previous meta-analysis, the mean concentration of PCT was found to be around 6 ng/mL in patients with Gram-positive and/or fungal infections, which is significantly higher than in healthy controls [12]. However, in Gram-negative infections, the PCT level was found to be even higher with its value being around 13 ng/mL, which indicates the level of induced PCT concentration differs among pathogens even in bacteremia [12]. Though the proposed optimal cutoffs varied greatly from 0.291 ng/mL to 16.9 ng/mL in our included studies, the results consistently indicated a higher level of PCT in Gram-negative infections than in Gram-positive and/or fungal infections [17–20, 22, 23, 26–28, 30, 31, 34, 40, 41]. Therefore, with algorithms based on staged cutoffs, e.g., 6 ng/mL for differentiation between Gram-positive (and/or fungal) infections and healthy controls and 13 ng/mL for differentiation between Gram-negative infections and Gram-positive (and/or fungal) infections, PCT was potentially helpful in differential diagnosis among bloodstream infections or sepsis arising from diverse pathogens [8]. However, it should be noted that the cutoffs should be carefully selected based on the population characteristics and assay techniques, because significant heterogeneity was identified between different clinical settings in our meta-analysis. Though our study failed to identify statistically significant differences in the diagnostic performances (thresholds and accuracies) of PCT either between different types of BSIs or between different states of culture positivity (culture positive or negative), there were nonsignificant trends indicating PCT could be more useful for diagnosing GNBSI in patients with bacterial infections and positive cultures than in their opposite conditions.

The metaregression results suggested the diagnostic accuracy was relatively low in patients with hematological malignancies (acute leukemia, lymphoma, and other hematologic malignancies), implicating unreliability of the PCT test for diagnosing GNBSI in patients with hematological malignancy. Noticing that the optimal cutoffs reported in these studies were 0.5–1.52 ng/m, which was fairly close to the cutoff used in discriminating bacterial infection from nonbacterial infection, patients with hematological malignancy could possibly lose part of the ability to respond to Gram-negative bacteria or their products [8]. Our results also identified a nonsignificant trend indicating PCT could be of greater value in sepsis patients than in patients without sepsis. However, it should be noted that PCT is reported to correlate with the severity of infection and the diagnostic accuracy could be therefore affected. Unfortunately, we were not able to evaluate the impact of severity of infection because few of the included studies documented PCT values along with individual severity [52]. As Gram-negative infections are usually associated with increased severity of diseases, the issue whether PCT concentration is affected by severity or pathogen remained to be further discussed [53].

Demographical and technical characteristics were also crucial aspects in clinical practice. Although excellent performance has been reported in some East Asian studies with both sensitivity and specificity being over 85%, our pooled results failed to identify a significant difference between East Asian and European population [22, 23]. In our subgroup analysis, we failed to get exact age and sex information from some of the studies, which hindered the analytical process going further [21, 26, 31, 33, 36, 37, 40, 41, 54]. Our results assessing the performance of different PCT assays were in line with previous studies which demonstrated equivalence among 3 different PCT assays (Kryptor, Vidas, or Elecsys/Cobas), as the threshold and accuracy were suggested consistent across these three tests in our study [55, 56]. A study comparing recent popular PCT assay systems showed the results from these systems correlated well, but their regression lines varied considerably. In future research, pre-experimental calibration could possibly help reduce heterogeneity when diagnostic tests using different assay systems were compared in a single study [57].

### 4.2. PCT in Comparison with Other Biomarkers

CRP and IL-6 were the markers most frequently compared with PCT, while the results were somehow inconsistent [17, 18, 21–24, 27, 30, 33–35, 38, 39]. This issue was further explored in our study by direct comparison; the findings suggested variation in diagnostic accuracy across different thresholds, which meant the diagnostic accuracy of PCT was superior to CRP at some certain thresholds while inferior at others, but no significant difference was found between PCT and IL-6. Under most circumstances, PCT should be recommended over CRP, as the overall diagnostic accuracy of PCT was higher than CRP. Though the diagnostic accuracy of IL-6 was found higher than PCT in some researches, the direct comparison failed to identify a statistical significance [39]. In clinical practice, IL-6 has potential in serving together with PCT as markers for GNBSI and researches are needed for comparative effectiveness of IL-6 under different clinical settings [33, 39].

Endotoxemia was another widely investigated marker for GNBSI and was also systematically reviewed for prediction of GNBSI [58]. The pooled DORs of endotoxemia were 3.2 and 5.8 in association with GNBSIs with *Escherichia coli* and those with *Pseudomonas aeruginosa*, which were both lower than the DORs of PCT, CRP, and IL-6 derived in our study [58]. However, because none of the studies assessed PCT and endotoxemia tests in the same population, direct comparison between PCT and endotoxemia was not feasible. Increased leukocyte count is also demonstrated in some researches as a feature of GNBSI and showed potential in differentiation between GNBSI and other types of bloodstream infection, but few studies analyzed the corresponding diagnostic indices, such as specificity, sensitivity, and AUC [59–65]. Additionally, promising results of TNF-*α* and IL-8 tests were reported in predicting GNBSI in abdominal sepsis patients, with AUCs being 0.912 and 0.999, sensitivities being 90.2% and 97.6%, and specificities being 87.5% and 100%, respectively [66]. Performance of prepsin and IL-10 in recognizing GNBSI was found superior to PCT in certain contexts, including adult patients after HSCT and children with hematology-oncology disease [35, 38]. Although these markers were found valuable in diagnosing GNBSI, the number of studies were not enough for a meta-analysis [66–73]. Alternatively, the use of comprehensive sets of markers, especially those correlated with severity of the disease, together with PCT may help improve its performance in recognizing GNBSI [22, 66].

### 4.3. Limitations

Our meta-analysis has several limitations. First, information on patients' medical condition is extremely limited. Patients with suspected BSI could have diverse comorbidities, while most studies only recorded comorbidities of interest, e.g., sepsis and hematological malignancy. Changes in patients' medical conditions could cause fluctuations in PCT level and therefore affect the diagnostic performance. Also, the PCT levels could be influenced by some drugs, such as antithymocyte globulin (ATG) [74]. Second, the timing of measurement was seldom mentioned in our included studies. Once triggered by toxins, PCT increases in a sigmoid manner, false negatives might take place at an early stage if toxins were not enough for triggering a surge in PCT levels [8, 10]. Third, since there were no established criteria for selecting the optimal cutoff in this diagnostic theme, 11 studies used ROC analysis to derive optimal cutoffs. A predefined cutoff could help in reducing the bias in sensitivity and specificity possibly caused by this data-driven method [42]. Additionally, in this present study, we were not able to calculate a specific cutoff for clinical use, because individual patient data on PCT concentration was not available in most studies.

## 5. Conclusions

PCT was helpful in recognizing Gram-negative bloodstream infection, but the results should be carefully interpreted with full knowledge of patients' medical condition. In patients with hematological malignancy, PCT should not be encouraged to be used as a marker for GNBSI. Also, results of PCT tests should be interpreted separately in adult and pediatric population. Though PCT showed a higher diagnostic odds ratio compared to CRP and IL-6, selection of the optimal biomarkers should be done carefully considering the required range of the sensitivity and specificity. In future research, features of medical context, demographics, and demands for sensitivity and specificity should be taken into consideration. Further prospective studies are warranted for comparisons between different clinical settings.

## Figures and Tables

**Table 1:** Characteristics of included studies.

Author	Year	Country	Setting	Medical contexts	Culture results	AUC for PCT (95% CI)	Episodes; patients	Male	Age^††^; age range (yrs)
Yan [41]	2018	China	ICU and EICU	Nosocomial pneumonia	GN (163), GP (139)	0.71^∗∗^	302; reported 345 BCs from 286 patients	60%^∗^	73.5 (62–82) ^∗^; >18^∗^
Yan [40]	2017	China	Multidepartments	Sepsis	GN (254), GP (202)	0.63 (0.58-0.68)	456; reported 524 BCs from 414 patients	61%^∗^	70 (59–80) ^∗^; >18^∗^
Xu [39]	2019	China	Hematology	Hematologic malignancy	GN (217), other hemocultures (179 GPs + 6 fungi + 9 mixed + 2708 negatives = 2902)^‡‡^	0.68 (0.64-0.72)	3118; 1115	60%	6.1; 1 mos–17.5 yrs
Xia [38]	2016	China	Hematology/Oncology Department	Fever^†^	GN (154), other hemocultures (2665, included negative cultures)	0.66 (0.59-0.72)	2819; reported 3023 BCs from 992 children	61%	NR; 0.1-17.5
Vincenzi [37]	2016	Italy	Medical Oncology Department	Malignancy (febrile patients with solid metastatic or locally advanced tumor)	GN (130), other hemocultures (45 GPs + 6 fungi = 51)	0.77^∗∗^	181; 181	55%^∗^	NR; 18-60 (34.6%), 61-70 (29.2%), >70 (36.2%)^∗^
Thomas [36]	2018	Germany	ICU	Sepsis (severe)	GN (815), other hemocultures (4043, included negative cultures)	0.72 (0.71-0.74)	4858; 4858	63%	70 (59-77); >18
Stoma [35]	2017	Belarus	Center of Hematology and Bone Marrow Transplantation	Malignancy, febrile neutropenia (after HSCT)	GN (30), other hemocultures (3 CMV + 1 Candida + 18 negatives = 22)	0.74 (0.57-0.87)	52; 52	46%	41 (28-51); 18-79
Shao [34]	2018	China	Multidepartments	Sepsis	GN (170), GP (209)	0.78^∗∗^	379; 379	60%	1; (4 d-13.3 yrs)
Prat [33]	2008	Spain	Hematology	Malignancy, febrile neutropenia	GN (5), other hemocultures (14 GPs + 38 negative cultures = 52)	0.86 (0.74-0.99)	57; 56	51%^∗^	47^∗^; (15-69) only one patient below 18^∗^
Oussalah [32]	2015	France	Multidepartments	Suspected BSI	GN (1067), other hemocultures (975 GPs + 401 other bacterial genera + 256 fungi + 30996 negatives = 34276)	0.75^∗∗^	35343; 35343	NR	49 (13-66); 0-102
Nishikawa [31]	2017	Japan	Multidepartments	Fever	GN rods (69), GP cocci (100)	0.87^∗∗^	169; 169	12%^∗^	66 (37-76) ^∗^; >18^∗^
Nakajima [30]	2014	Japan	Shock Trauma Center and Department of Respiratory Medicine	Sepsis	GN rods (6), GP cocci (8)	0.85^∗∗^	14; 14	78%	61.8 (11.2); >18
Luo [29]	2019	China	Hematology	Malignancy, febrile neutropenia	GN (268), other hemocultures (GP + fungi = 107)	0.70 (0.67-0.74)	375; reported 1466 BCs from 396 patients	61%^∗^	38.0 (27-52) ^∗^; >14^∗^
Liu [28]	2017	China	Multidepartments	Sepsis	GN (91), GP (56)	0.73 (0.65-0.81)	147; 147	67%	59.3 (2.3); >18
Li [27]	2016	China	Multidepartments	Sepsis	GN (158), GP (140)	0.79 (0.74-0.84)	298; 298	65%	64.1 (19.4); >18
Leli [26]	2015	Italy	Multidepartments	Sepsis	GN (345), GP (217)	0.77 (0.73-0.81)	562; 562	59%^∗^	74 (62-83); >18^∗^
Kok [25]	2019	China	Multidepartments	Fever (suspected BSI)	GN (228), other hemocultures (GP + unspecified bacteremia = 658)	NR	886; 886	NR	NR; >18
Koivula [24]	2011	Finland	Hematology	Malignancy, febrile neutropenia	GN (10), other hemocultures (75, including negative cultures)^‡^	0.77^∗∗^	85; 66	70%	56; 18-70
Gao [23]	2017	China	Multidepartments	Sepsis	GN (47), GP (45)	0.97^∗∗^	92; 92	58%	54.0 (8.0); 40-78
Fu [22]	2012	China	ICU	Sepsis	GN (23), Candida (20)	0.90^∗∗^	43; 43	63%	62.4 (2.8); >18
Fleischhack [21]	2000	Germany	Pediatric Hematology/Oncology Ward	Malignancy, febrile neutropenia	GN (13), other hemocultures (60 FUO + 28 localized infections + 13 pneumonias + 7 GPs + 1 fungus = 109)	NR	122; 51	61%	9; 0.7-31.8
Charles [20]	2008	France	ICU	Sepsis	GN (52), GP (45)	0.79 (0.71-0.88)	97; 92	64%	64.8 (15.3); >18
Cabral [19]	2018	Portugal	Burn unit	Sepsis (in burn patients)	GN (75), GP (114)	0.69 (0.61-0.77)	189; 189	59%	66 (GN group), 69 (GP group); >18
Brodska [18]	2013	Czech Republic	ICU	Sepsis	GN (78), GP and fungus (88)	0.871^∗∗^	166; 166	42%	64.5 (55-76); >18
Bilgili [17]	2018	Turkey	ICU	Sepsis	GN (76), GP (48)	0.80 (0.72-0.89)	124; 124	49%	56.3 (19.7); >18

^∗^Characteristics reported in a larger population in the original studies: Yan 2018 (286), Yan 2017 (414), Vincenzi (431), Prat (61), Nishikawa (852), Leli (1949), Luo (396). ^†^Febrile patients included positive blood culture sepsis, clinical sepsis, nonsepsis infection, viral infection, and systemic fungal infection. ^‡^Other febrile episodes included fever episodes with no bacteremia and Gram-positive bacteremia. ^∗∗^95% CI not available. ^††^Ages were expressed as mean (SD) or median (IQR) or median. ^‡‡^Proportion of mixed culture results was less than 0.5%. AUC: area under the receiver operating characteristic curve; CI: confidence interval; yrs: years; mos: months; ICU: intensive care unit; EICU: emergency intensive care unit; IQR: interquartile range; GN: Gram-negative; GP: Gram-positive; HSCT: hematopoietic stem cell transplantation; FUO: fever of unknown origin; NR: not reported.

**Table 2:** Subgroup and metaregression analysis for PCT.

Covariate	Category	Study	n	AUC (95% CI)	Pooled sensitivity (95% CI)	Pooled specificity (95% CI)	*p*1 (for threshold)	*p*2 (for accuracy)
Covariates of medical contexts								
BSI type	GN VS GP	11	2639	0.82 (0.79-0.85)	0.77 (0.70-0.83)	0.74 (0.63-0.83)	0.157	0.157
	Others	14	48294	0.78 (0.74-0.81)	0.65 (0.58-0.71)	0.77 (0.72-0.81)		
Culture	Positive culture only	16	3593	0.81 (0.78-0.85)	0.75 (0.69-0.80)	0.75 (0.67-0.82)	0.317	0.107
	Others	9	47340	0.75 (0.71-0.79)	0.61 (0.52-0.69)	0.76 (0.70-0.81)		
Sepsis status	Sepsis only	13	7424	0.82 (0.79-085)	0.76 (0.70-0.81)	0.75 (0.66-0.82)	0.403	0.157
	Others	12	43509	0.76 (0.72-0.80)	0.63 (0.55-0.71)	0.77 (0.70-0.82)		
Hematological malignancy	Hematological malignancy only	7	6628	0.69 (0.65-0.73)	0.52 (0.46-0.59)	0.79 (0.74-0.83)	0.273	0.032^∗^
	Others	18	44305	0.80 (0.77-0.84)	0.75 (0.70-0.79)	0.74 (0.67-0.80)		
Febrile neutropenia status	Febrile neutropenia only	5	691	0.69 (0.65-0.73)	0.57 (0.50-0.64)	0.81 (0.76-0.85)	1.000	0.752
	Others	20	50242	0.80 (0.76-0.83)	0.73 (0.67-0.78)	0.74 (0.68-0.80)		
Covariates of demographical features								
Region	Europe	12	41836	0.77 (0.74-0.81)	0.69 (0.64-0.73)	0.78 (0.72-0.83)	0.317	0.752
	East Asia	13	9097	0.80 (0.76-0.83)	0.74 (0.64-0.81)	0.74 (0.65-0.81)		
Setting	ICU only	6	5590	0.82 (0.78-0.85)	0.76 (0.69-0.82)	0.76 (0.63-0.86)	0.237	0.221
	Others	19	45343	0.78 (0.75-0.82)	0.69 (0.62-0.75)	0.76 (0.70-0.81)		
Population	Adult only	19	8777	0.81 (0.77-0.84)	0.74 (0.69-0.78)	0.77 (0.72-0.82)	0.043^∗^	0.048^∗^
	Others	6	42156	0.74 (0.70-0.77)	0.60 (0.48-0.70)	0.73 (0.67-0.78)		
Other covariates								
PCT assay method	VIDAS	8	7538	0.75 (0.71-0.79)	0.70 (0.64-0.76)	0.70 (0.60-0.79)	0.091	0.150
	KRYPTOR	5	35957	0.76 (0.72-0.79)	0.75 (0.68-0.81)	0.65 (0.58-0.72)		
	ECLIA	8	4355	0.87 (0.84-0.90)	0.75 (0.59-0.86)	0.84 (0.77-0.90)		
Sample	Serum	16	14610	0.79 (0.75-0.82)	0.70 (0.62-0.76)	0.75 (0.68-0.80)	0.129	0.192
	Plasma	9	36323	0.79 (0.75-0.82)	0.73 (0.68-0.77)	0.78 (0.68-0.85)		

Subgroup and metaregression analysis of covariates. *p*1: *p* value for likelihood ratio test assessing impact of covariates on threshold; *p*2: *p* value for likelihood ratio test assessing impact of covariates on accuracy. AUC: area under summary receiver operating characteristic curve; CI: confidence interval; BSI: bloodstream infection; GN: Gram-negative bloodstream infection; GP: Gram-positive bloodstream infection; PCT: procalcitonin; ECLIA: electrochemiluminescence immunoassay; ICU: intensive care unit. ^∗^*p* < 0.05.

**Table 3:** Diagnostic performance of CRP and IL-6.

Author	Year	Optimal cutoff^∗^	AUC (95% CI)	Sensitivity	Specificity	GN episodes	Total episodes
CRP in 7371 episodes							
Bilgili [17]	2018	51.8	0.61 (0.512–0.716)	82.9	58.3	76	124
Brodska [18]	2013	86.2	0.705 (NR)	61.5	54.5	78	166
Fleischhack [21]	2000	50	NR (NR)	75.0	73.2	13	122
Fu [22]	2012	116	0.82 (NR)	82.6	75.0	23	43
Gao [23]	2017	74.65	0.953 (NR)	93.6	91.1	47	92
Koivula [24]	2011	100	NR (NR)	54.5	63.6	11	88
Li [27]	2016	59.25	0.678 (0.541–0.814)	74.7	65.7	158	298
Nakajima [30]	2014	475	0.738 (0.454-0.100	100.0	57.1	6	13
Prat [33]	2008	135	0.665 (0.475–0.856)	100.0	51.0	5	57
Shao [34]	2018	16	0.785 (NR)	62.7	87.0	170	379
Stoma [35]	2017	165	0.707 (0.564–0.825)	40.0	91.0	30	52
Xia [38]	2016	40	0.596 (0.527–0.666)	51.2	63.2	154	2819
Xu [39]	2019	90	0.557 (0.516–0.597)	44.7	80.0	217	3118
Pooled results with 95% CI (for CRP)			0.78 (0.74–0.81)	0.72 (0.59–0.81)	0.72 (0.63–0.79)		
Pooled results with 95% CI (for PCT)			0.85 (0.81–0.87)	0.73 (0.63–0.81)	0.81 (0.76–0.85)		
IL-6 in 3455 episodes							
Fleischhack [21]	2000	20	NR (NR)	44.4	80.2	13	122
Fu [22]	2012	186.5	0.82 (NR)	82.6	80	23	43
Gao [23]	2017	171.65	0.925 (NR)	93.6	90.5	47	92
Shao [34]	2018	75.7	0.74 (NR)	78.2	69.6	170	379
Xia [38]	2016	279.4	0.686 (0.622-0.750)	56.9	75.4	154	2819
Pooled results with 95% CI (for IL-6)			0.83 (0.80-0.86)	0.76 (0.58–0.88)	0.79 (0.71–0.85)		
Pooled results with 95% CI (for CRP)			0.85 (0.81–0.87)	0.75 (0.56–0.87)	0.80 (0.68–0.88)		
Pooled results with 95% CI (for PCT)			0.87 (0.84–0.90)	0.80 (0.60–0.91)	0.82 (0.72–0.89)		

^∗^mg/L for CRP and pg/mL for IL-6; AUC: area under receiver operating characteristic curve; GN: Gram-negative; NR: not reported.

## Data Availability

The dataset can be requested by sending an email to the corresponding author.

## Supplementary Materials

Supplementary 1 Supplementary Table S1. PRISMA 2009 checklist.

Supplementary 2 Supplementary Table S2. Tailored QUADAS-2 tool.

Supplementary 3 Supplementary Table S3. Discarded signaling question.

Supplementary 4 Supplementary Table S4. Additional study characteristics.

Supplementary 5 Supplementary Figure S1. Methodological quality summary. Green spots with “+” indicate low risk of bias; yellow spots with “?” indicate unclear risk of bias; red spots with “-” indicate high risk of bias.

Supplementary 6 Supplementary Figure S2. Methodological quality graph.

Supplementary 7 Supplementary Figure S3. Pooled diagnostic odds ratio of PCT for recognizing GNBSI in contexts of bloodstream infection (BSI).

Supplementary 8 Supplementary Figure S4. Fagan nomogram for PCT in recognizing GNBSI.

Supplementary 9 Supplementary Figure S5. Results of Deeks' funnel plot asymmetry test for PCT in recognizing GNBSI.
