# Supplementary material for: Diagnostic Accuracy of Procalcitonin Compared to C-Reactive Protein and Interleukin 6 in Recognizing Gram-Negative Bloodstream Infection: A Meta-Analytic Study
Source: Dis Markers. 2020 Jan 23;2020:4873074. doi: 10.1155/2020/4873074 (PMC7008263; doi:10.1155/2020/4873074)
Supplement: Supplementary 2 — Supplementary Table S2. Tailored QUADAS-2 tool. [file 4873074.f2.docx]

**Supplementary Table S2. Tailored QUADAS-2 tool.**

| **Domain** | **Item** | **Questions and judging criteria** |
| --- | --- | --- |
| Patient selection | Signaling question | 1. Was a consecutive or random sample of patients enrolled?  2. Was a case-control design avoided?  3. Did the study avoid inappropriate exclusions?  The criteria for judgment is described in the guidance for QUADAS-2 [1]. |
|  | Risk of bias | Could the selection of patients have introduced bias?  Low risk: all answers to the questions in this domain are ‘yes’.  Unclear: information not enough for judging as high or low risk.  High risk: included healthy control without suspected blood stream infection (BSI). |
|  | Concerns regarding applicability | Is there concern that the included patients do not match the review question?  Low: patients were suspected to have bloodstream infections [2].  Unclear: information not enough for judging as high or low concern.  High: patients were highly unlikely to develop bloodstream infections. |
| Index test | Signaling question | If a threshold was used, was it pre-specified?  Yes: the cutoff values used was pre-specified by the respective manufacturers or guidelines.  No: the cutoff values were the optimal cutoff value derived from the ROC curve in a data-driven way [4].  Unclear: information not enough for judging as ‘yes’ or ‘no’. |
|  | Risk of bias | Could the conduct or interpretation of the index test have introduced bias?  Low: the answer to the signaling question in this domain is ‘yes’ or the sample size in each side is larger than 100 [4].  Unclear: the answer was no but the total number of included patient is more than 200.  High: situations other than ‘low’ or ‘unclear’.^1^ |
|  | Concerns regarding applicability | Is there concern that the index test, its conduct, or interpretation differ from the review question?  Low: employed a quantitative serum PCT measurement method.  Unclear: information not enough for judging as high or low concern.  High: PCT level was not measured in serum or not strictly quantitative. |
| Reference standard | Signaling question | 1. Is the reference standard likely to correctly identify the target condition?  Yes: culture process and the identification process of pathogens should be performed at an externally accredited platform of kit under standard protocol, e.g. BACTEC 9240 and VITEK II.  No: used unaccredited platforms or protocols.  Unclear: information not enough for judging as ‘yes’ or ‘no’.  2. Were the reference standard results interpreted without knowledge of the results of the index test?  Yes: the person undertaking reference test was unaware of the results of the index tests, or the result comes from relative objective methods, e.g. VITEK II and matrix-assisted laser desorption/ionization-time-of-flight mass spectrometry.  No: same person performed both tests, or the results of the index tests were known.  Unclear: information not enough for judging as ‘yes’ or ‘no’. |
|  | Risk of bias | Could the reference standard, its conduct, or its interpretation have introduced bias?  Low: the answers to all signaling questions in this domain should be yes  Unclear: information not enough for judging as ‘low’ or ‘high’.  High: reference standard results were interpreted with knowledge of the results of the index test, then scored high |
|  | Concerns regarding applicability | Is there concern that the target condition as defined by the reference standard does not match the review question?  Low: used techniques based on culture process to gain the results, like Gram-stain or MALDI-TOF.  Unclear: information not enough for judging as ‘low’ or ‘high’.  High: used techniques not based on culture method. |
| Flow and timing | Signaling questions | 1. Was there an appropriate interval between index test(s) and reference standard?  Yes: 12 or fewer hours of interval between the index test and reference test [5].  No: the time interval between index test and reference standard collection was > 12 hours [5].  Unclear: information not enough for judging as ‘yes’ or ‘no’.  2. Did patients receive the same reference standard?  The criteria for judgment is described in the guidance for QUADAS-2 [1].^1^  3. Were all patients included in the analysis?  The criteria for judgment is described in the guidance for QUADAS-2 [1].^1^ |
|  | Risk of bias | Could the patient flow have introduced bias?  Low: all answers to the signaling questions in this domain are ‘yes’.  Unclear: information not enough for judging as ‘low’ or ‘high’.  High: any ‘no’ in the answers to the signaling questions in this domain. |

**Reference:**

1. Whiting PF, Rutjes AW, Westwood ME, Mallett S, Deeks JJ, Reitsma JB , et al. QUADAS-2: a revised tool for the quality assessment of diagnostic accuracy studies. Ann Intern Med 2011; 155:529-536.

2. Baron EJ, Miller JM, Weinstein MP, Richter SS, Gilligan PH, Thomson RB, Jr. , et al. A guide to utilization of the microbiology laboratory for diagnosis of infectious diseases: 2013 recommendations by the Infectious Diseases Society of America (IDSA) and the American Society for Microbiology (ASM)(a). Clinical infectious diseases : an official publication of the Infectious Diseases Society of America 2013; 57:e22-e121.

3. Dandona P, Nix D, Wilson MF, Aljada A, Love J, Assicot M , et al. Procalcitonin increase after endotoxin injection in normal subjects. The Journal of clinical endocrinology and metabolism 1994; 79:1605-1608.

4. Leeflang MM, Moons KG, Reitsma JB, Zwinderman AH. Bias in sensitivity and specificity caused by data-driven selection of optimal cutoff values: mechanisms, magnitude, and solutions. Clin Chem 2008; 54:729-737.

5. Oussalah A, Ferrand J, Filhine-Tresarrieu P, Aissa N, Aimone-Gastin I, Namour F , et al. Diagnostic Accuracy of Procalcitonin for Predicting Blood Culture Results in Patients with Suspected Bloodstream Infection. Medicine (United States) 2015; 94:e1774.
