# Supplementary material for: Diagnostic Accuracy of Procalcitonin Compared to C-Reactive Protein and Interleukin 6 in Recognizing Gram-Negative Bloodstream Infection: A Meta-Analytic Study
Source: Dis Markers. 2020 Jan 23;2020:4873074. doi: 10.1155/2020/4873074 (PMC7008263; doi:10.1155/2020/4873074)
Supplement: Supplementary 3 — Supplementary Table S3. Discarded signaling question. [file 4873074.f3.docx]

**Supplementary Table S3. Discarded signaling question in Quadas-2 tool.**

| **Domain** | **Signaling question** | **Reason for exclusion** |
| --- | --- | --- |
| Index test – Risk of bias | Were the index test results interpreted without knowledge of the results of the reference standard? | The techniques for measurement of blood PCT level were objective index tests, the results were independent of subjective judgements. |
