# Supplementary material for: Diagnostic Accuracy of Procalcitonin Compared to C-Reactive Protein and Interleukin 6 in Recognizing Gram-Negative Bloodstream Infection: A Meta-Analytic Study
Source: Dis Markers. 2020 Jan 23;2020:4873074. doi: 10.1155/2020/4873074 (PMC7008263; doi:10.1155/2020/4873074)
Supplement: Supplementary 4 — Supplementary Table S4. Additional study characteristics. [file 4873074.f4.docx]

**Supplementary Table S4. Additional study characteristics**

| Author | Year | Country | Study design | Sample | Procalcitonin assay | PCT check time | Cutoff (ng/mL) | TP | FP | FN | TN | SEN (%) | SPEC (%) | GN prevalance (%) |
| --- | --- | --- | --- | --- | --- | --- | --- | --- | --- | --- | --- | --- | --- | --- |
| Yan^41^ | 2018 | China | Retrospective | serum | BRAHMS-VIDAS | NR | 0.465* | 139 | 71 | 24 | 68 | 85.4 | 49.2 | 55.7% |
|  |  |  |  |  |  |  | 1.05 | 114 | 60 | 49 | 79 | 70.0 | 56.8 |  |
|  |  |  |  |  |  |  | 5.4 | 67 | 27 | 96 | 112 | 40.8 | 80.3 |  |
|  |  |  |  |  |  |  | 10.16 | 54 | 18 | 109 | 121 | 33.1 | 87.1 |  |
|  |  |  |  |  |  |  | 15.9 | 39 | 14 | 124 | 125 | 23.8 | 90.2 |  |
| Yan^40^ | 2017 | China | Retrospective | serum | BRAHMS-VIDAS | NR | 0.495* | 184 | 99 | 70 | 103 | 72.4 | 51.0 | 54.0% |
|  |  |  |  |  |  |  | 5.05 | 100 | 53 | 154 | 149 | 39.4 | 73.8 |  |
|  |  |  |  |  |  |  | 10.335 | 79 | 40 | 175 | 162 | 31.1 | 80.2 |  |
|  |  |  |  |  |  |  | 15 | 66 | 32 | 188 | 170 | 26.0 | 84.2 |  |
| Xu^39^ | 2019 | China | Prospective | serum | ROCHE-E411 | the onset of fever | 0.58 | 97 | 580 | 120 | 2321 | 44.8 | 80.0 | 7.0% |
| Xia^38^ | 2016 | China | Retrospective | serum | BRAHMS-VIDAS | at admission or ≤ 24 h of fever onset | 0.5* | 78 | 682 | 76 | 1983 | 50.4 | 74.4 | 5.5% |
|  |  |  |  |  |  |  | 1 | 50 | 405 | 104 | 2260 | 32.6 | 84.8 |  |
|  |  |  |  |  |  |  | 2 | 29 | 320 | 125 | 2345 | 18.6 | 88.0 |  |
| Vincenzi^37^ | 2016 | Italy | Retrospective | plasma | BRAHMS- KRYPTOR | NR | 1.52 | 94 | 15 | 36 | 36 | 72.1 | 70.1 | 71.8 % |
| Thomas^36^ | 2018 | Germany | Retrospective | serum | BRAHMS-VIDAS | ≤ 24 h after the onset of severe sepsis | 10 | 562 | 1415 | 253 | 2628 | 69.0 | 65.0 | 16.8 % |
| Stoma^35^ | 2017 | Belarus | Prospective | serum | BRAHMS-VIDAS | ≤ 4 h after the onset of febrile neutropenia | 1.5 | 19 | 3 | 11 | 19 | 62.0 | 88.0 | 57.7% |
| Shao^34^ | 2018 | China | Prospective | serum | ROCHE-E411 | NR | 0.291 | 110 | 36 | 60 | 173 | 64.7 | 82.6 | 44.9% |
| Prat^33^ | 2008 | Spain | Prospective | serum | BRAHMS-PCT LIA | ≤ 4 h after the onset of febrile neutropenia | 0.1 | 3 | 9 | 2 | 43 | 100 | 38.5 | 8.8% |
|  |  |  |  |  |  |  | 0.3 | 5 | 32 | 0 | 20 | 100 | 51.9 |  |
|  |  |  |  |  |  |  | 0.5 | 3 | 10 | 2 | 42 | 60.0 | 80.8 |  |
|  |  |  |  |  |  |  | 1* | 3 | 7 | 2 | 45 | 60.0 | 86.5 |  |
|  |  |  |  |  |  |  | 2 | 2 | 3 | 3 | 49 | 40.0 | 94.2 |  |
| Oussalah^32^ | 2015 | France | Retrospective | plasma | BRAHMS- KRYPTOR | NR | 0.6 | 821 | 12778 | 246 | 21498 | 76.9 | 62.7 | 3.0% |
| Nishikawa^31^ | 2017 | Japan | Retrospective | plasma | ROCHE-COBAS | NR | 8.8 | 42 | 9 | 27 | 91 | 61.0 | 91.0 | 40.8% |
| Nakajima^30^ | 2014 | Japan | Prospective | plasma | ROCHE-E411 | NR | 16.9 | 5 | 1 | 1 | 7 | 85.7 | 83.3 | 42.9% |
| Luo^29^ | 2019 | China | Retrospective | serum | ROCHE-E601 | NR | 0.56 | 148 | 25 | 120 | 82 | 55.2 | 76.6 | 71.5% |
| Liu^28^ | 2017 | China | Retrospective | plasma | BRAHMS- KRYPTOR | ≤ 1 h after the clinical diagnosis of sepsis | 2.1 | 76 | 29 | 15 | 27 | 83.5 | 48.2 | 61.9 % |
| Li^27^ | 2016 | China | Retrospective | serum | BRAHMS-VIDAS | ≤ 24 h after the clinical diagnosis of sepsis | 0.5 | 135 | 68 | 23 | 72 | 85.4 | 51.4 | 53.0% |
|  |  |  |  |  |  |  | 1.01 | 121 | 50 | 37 | 90 | 76.6 | 64.3 |  |
|  |  |  |  |  |  |  | 2.06 | 109 | 36 | 49 | 104 | 69.0 | 74.3 |  |
|  |  |  |  |  |  |  | 2.44* | 108 | 32 | 50 | 108 | 68.4 | 77.1 |  |
|  |  |  |  |  |  |  | 5.08 | 92 | 24 | 66 | 116 | 58.2 | 82.9 |  |
|  |  |  |  |  |  |  | 10.15 | 71 | 15 | 87 | 125 | 44.9 | 89.3 |  |
| Leli^26^ | 2015 | Italy | Prospective | {Nakajima, 2014 #154} serum | BRAHMS-VIDAS | ≤ 24 h after the clinical diagnosis of sepsis | 10.8 | 207 | 39 | 138 | 178 | 60.0 | 82.0 | 61.4 % |
| Kok^25^ | 2019 | China | Retrospective | serum | BRAHMS-VIDAS | ≤ 24 h after presumptive diagnosis of BSI | 3.9 | 153 | 138 | 75 | 520 | 67.1 | 79.0 | 25.7% |
| Koivula^24^ | 2011 | Finland | Prospective | plasma | BRAHMS-PCT-Q | ≤ 24 h after the onset of neutropenic fever | 0.5* | 7 | 17 | 3 | 58 | 70.0 | 77.0 | 11.8% |
|  |  |  |  |  |  |  | 2 | 4 | 3 | 6 | 72 | 40.0 | 95.9 |  |
|  |  |  |  |  |  |  | 10 | 3 | 0 | 7 | 75 | 30.0 | 100.0 |  |
| Gao^23^ | 2017 | China | Prospective | serum | ROCHE-ECL | NR | 8.35 | 45 | 3 | 2 | 42 | 96.8 | 93.5 | 51.1% |
| Fu^22^ | 2012 | China | Prospective | serum | ROCHE-E170 | NR | 8.06 | 20 | 3 | 3 | 17 | 87.0 | 85.0 | 53.5% |
| Fleischhack^21^ | 2000 | Germany | Retrospective | plasma | BRAHMS-PCT LIA | ≤ 24 h after the onset of fever | 0.3 | 10 | 61 | 3 | 48 | 80.0 | 44.0 | 10.7 % |
|  |  |  |  |  |  |  | 0.5* | 8 | 16 | 5 | 93 | 60.0 | 85.0 |  |
|  |  |  |  |  |  |  | 1 | 7 | 3 | 7 | 106 | 50.0 | 97.0 |  |
|  |  |  |  |  |  |  | 5 | 5 | 11 | 8 | 98 | 40.0 | 90.0 |  |
| Charles^20^ | 2008 | France | Retrospective | plasma | BRAHMS-KRYPTOR | at the onset of suspected BSI | 16 | 39 | 8 | 13 | 37 | 75.0 | 82.2 | 53.6 %* |
| Cabral^19^ | 2018 | Portugal | Retrospective | serum | BRAHMS-KRYPTOR | NR | 0.57 | 47 | 36 | 28 | 78 | 63.0 | 68.0 | 39.7 %* |
| Brodska^18^ | 2013 | Czech Republic | Retrospective | plasma | COBAS-ECLIA | NR | 0.5 | 74 | 61 | 4 | 27 | 94.9 | 30.7 | 47.0 %* |
|  |  |  |  |  |  |  | 5 | 55 | 20 | 23 | 68 | 70.5 | 77.3 |  |
|  |  |  |  |  |  |  | 15* | 59 | 11 | 19 | 77 | 75.1 | 87.8 |  |
| Bilgili^17^ | 2018 | Turkey | Retrospective | serum | BRAHMS-VIDAS | NR | 1.3 | 54 | 8 | 22 | 40 | 71.1 | 83.3 | 61.3 %* |

*, optimal cutoff in reported multiple cutoffs.
