# Supplementary figures and images for: Diagnostic Accuracy of Procalcitonin Compared to C-Reactive Protein and Interleukin 6 in Recognizing Gram-Negative Bloodstream Infection: A Meta-Analytic Study
Source: Dis Markers. 2020 Jan 23;2020:4873074. doi: 10.1155/2020/4873074 (PMC7008263; doi:10.1155/2020/4873074)

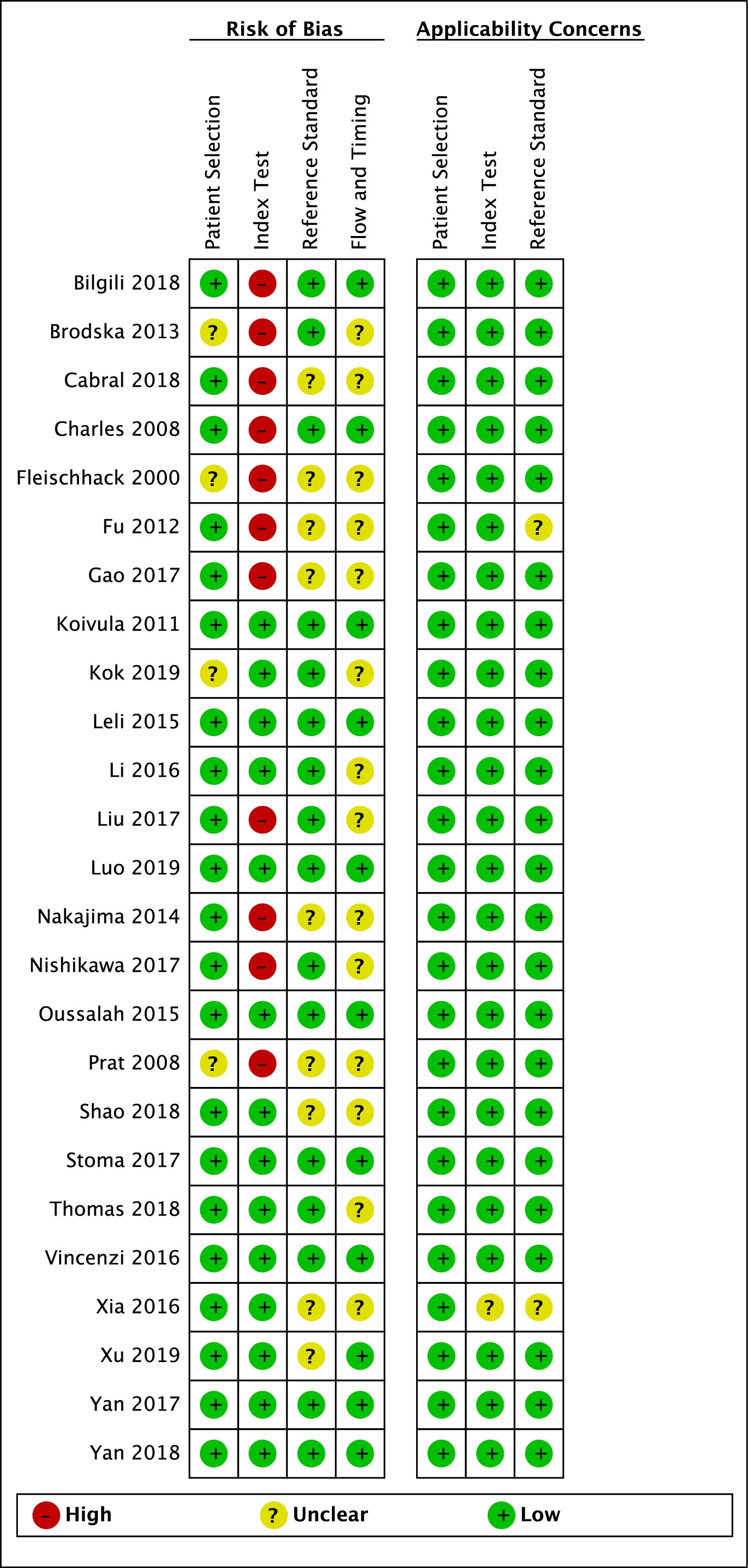

Supplement: Supplementary 5 — Supplementary Figure S1. Methodological quality summary. Green spots with “+” indicate low risk of bias; yellow spots with “?” indicate unclear risk of bias; red spots with “-” indicate high risk of bias. [file 4873074.f5.jpg]

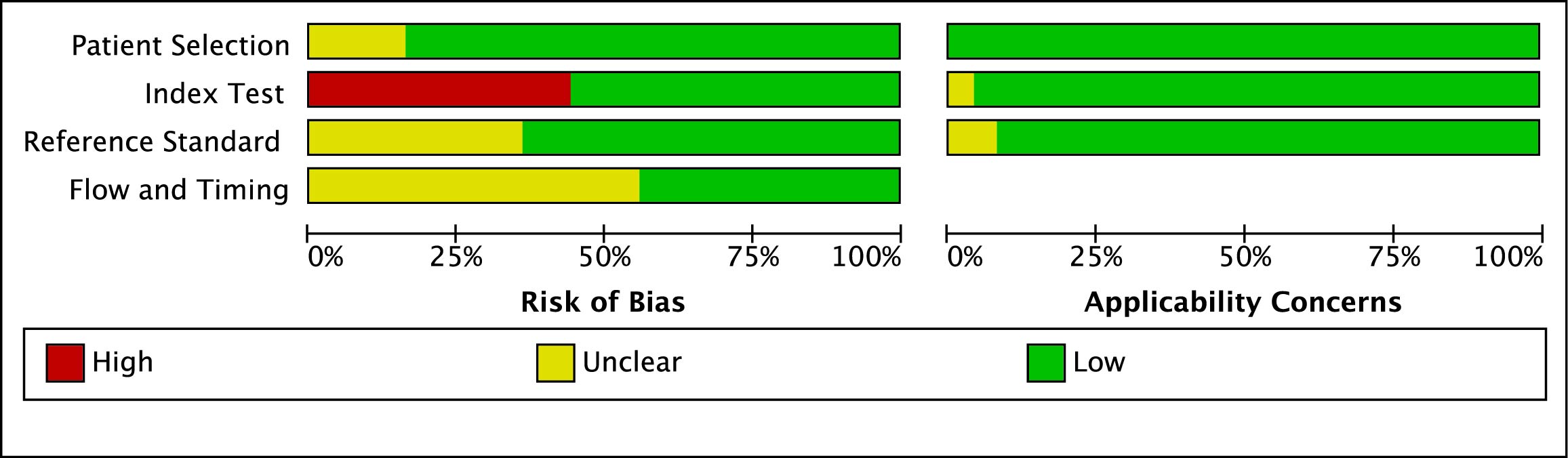

Supplement: Supplementary 6 — Supplementary Figure S2. Methodological quality graph. [file 4873074.f6.jpg]

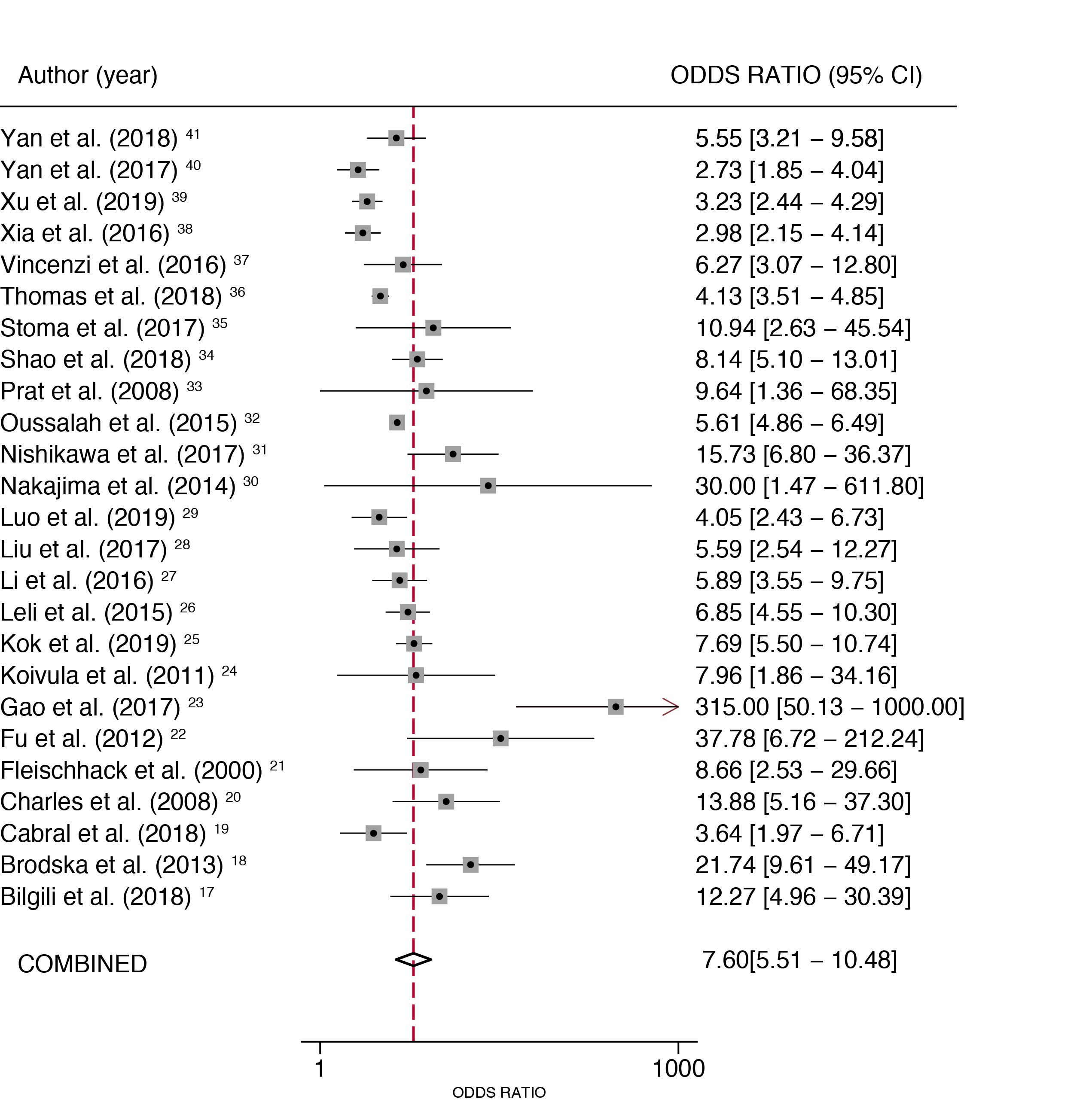

Supplement: Supplementary 7 — Supplementary Figure S3. Pooled diagnostic odds ratio of PCT for recognizing GNBSI in contexts of bloodstream infection (BSI). [file 4873074.f7.jpg]

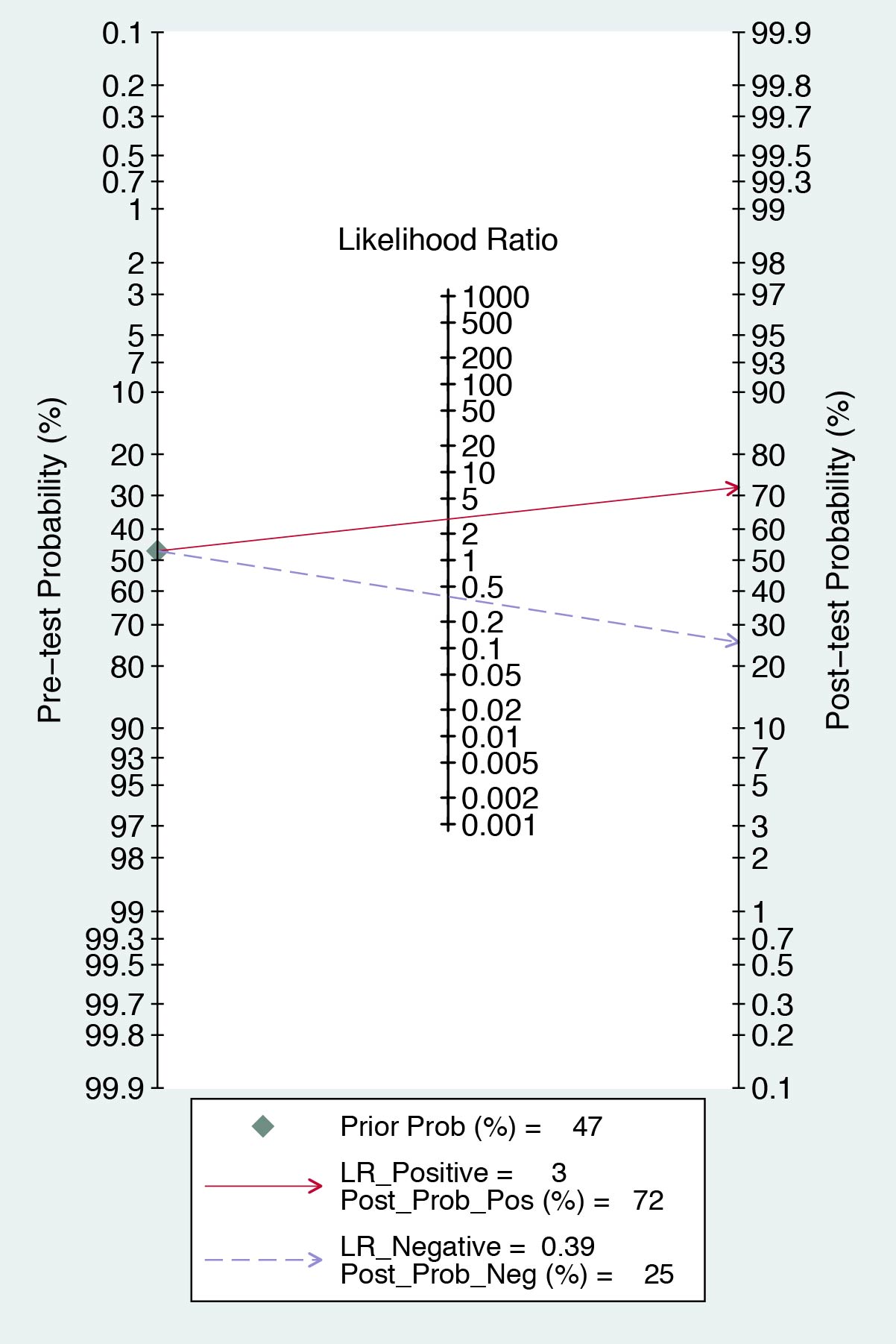

Supplement: Supplementary 8 — Supplementary Figure S4. Fagan nomogram for PCT in recognizing GNBSI. [file 4873074.f8.jpg]

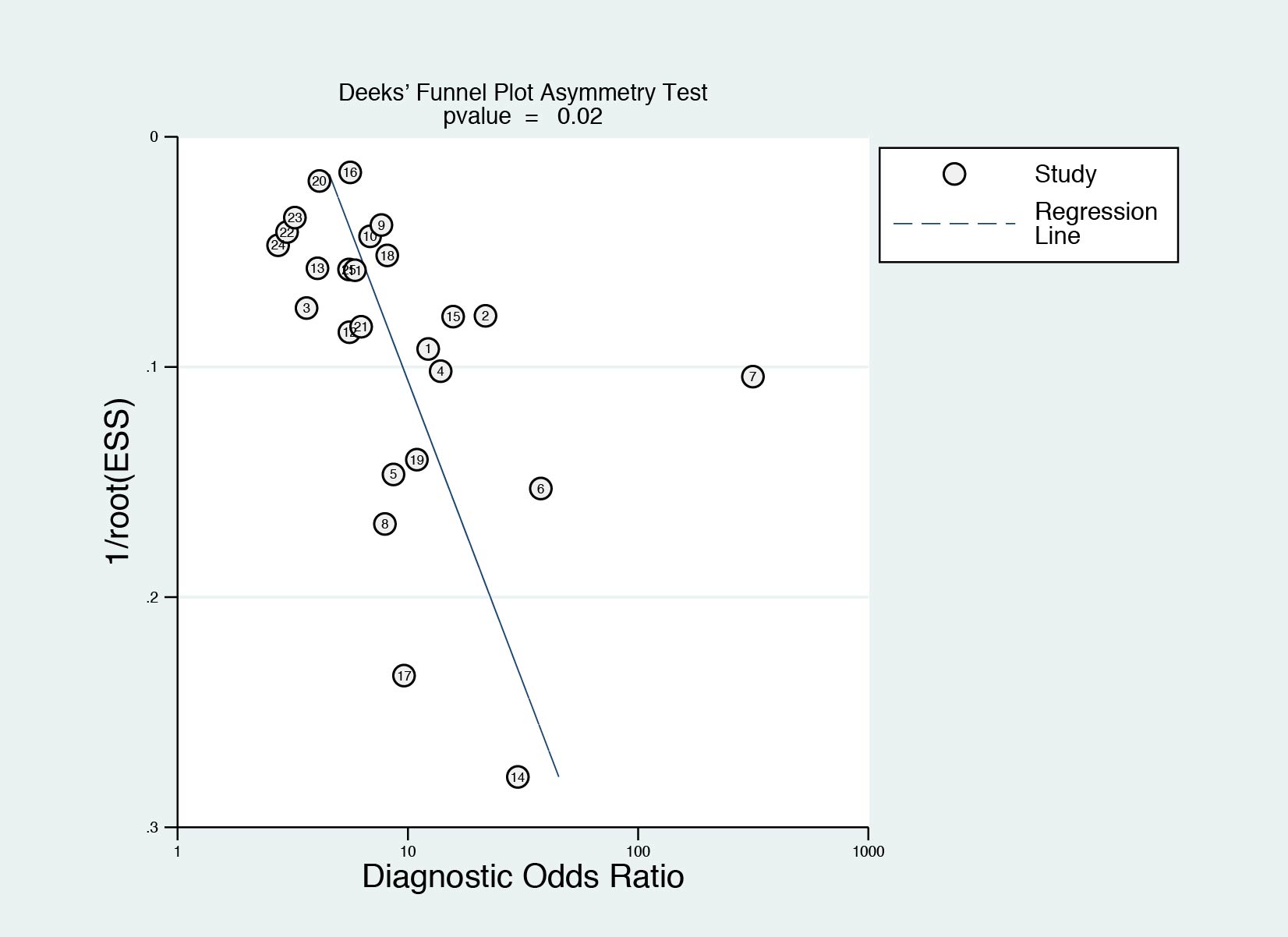

Supplement: Supplementary 9 — Supplementary Figure S5. Results of Deeks' funnel plot asymmetry test for PCT in recognizing GNBSI. [file 4873074.f9.jpg]
